# The Following Is a Correct Copy of the London Testimonial in Favour of Vaccine Inoculation

**Published:** 1801-09

**Authors:** 


### *The following is a correct Copy of the London Testimonial in Favour of Vaccine Inoculation.*

MANY unfounded reports having been circulated, which have a tendency to prejudice the public against the Inoculation of the Cow-pox; we, the underfigned phyficians and furgeons, think it our duty to declare our opinion, that thofe perfons who have had the Cow-pox, are perfectly fecure from the future infection of the Small-pox.

We alfo declare, that the inoculated Cow-pox is a much milder and fafer difeafe than the inoculated Small-pox.

Since our laft Report, the fignatures of the following Phyficians and Surgeons have been added.

Dr. Pitcairn
Dr. Latham
Dr. J. Mayo
Dr. Hunter
Dr. Pemberton
Dr. Frampton
Dr. Fearon
Dr. Cooke
Dr. William Hamilton
Dr. Pinckard
Dr. Elliot
Dr. Babington
Sir Walter Farquhar, Bart.
Dr. Yelloly
Dr. Clarke
Dr. Poignand
Dr. Maton
Dr. Hooper
Dr. Bancroft
Dr. Hawes
Dr. Haighton
Dr. Boys
Dr. Walfhman

### SURGEONS.

Mr. Addington
Mr. Aikin
Mr. Anderfon
Mr. Andree
Mr. Blifs
Mr. Brickenden
Mr. Thomas Brown
Mr. Bureau
Mr. James Burrows
Mr. Cairncrofs
Mr. Chamberlaine
Mr. Charlton
Mr. Clutterbuck
Mr. Coleman
Mr. Cribb
Mr. John Curtis
Mr. L. I. Curtis
Mr. William Curtis
Mr. Dale
Mr. John Davies
Mr. Douglas
Mr. Dowers
Mr. Dunn
Mr. Dyfon
Mr. Foot
Mr. Murray Forbes
Mr. William Forbes
Mr. Fofter
Mr. Gaitfkell
Mr. Gardner
Mr. Gib
Mr. Thomas Goodwin
Mr. Grenville
Mr. Griftock

Mr.

Mr. Harris
Mr Hayes
Mr. J. W. Hill
Mr. Hogben
Mr. Hole
Mr. Hollings
Mr. William Holt
Mr. Home
Mr. Jeaffrefon
Mr. Johnfon
Mr. Jordan
Mr. Key
Mr. David Lewis
Mr. Lewthwaite
Mr. Luxmore
Mr. Maffie
Mr. Maule
Mr. Meffiter
Mr. Mofs
Mr. O'Connor
Mr. Owen
Mr. James Parkinfon
Mr. Paternofter
Mr. Perfect
Mr. Phillips
Mr. Platt
Mr. Porter
Mr. Ramfden
Mr. Reid
Mr. Ricardo
Mr. Seagram
Mr. Seares
Mr. Sharp
Mr. Thomas Sheldon
Mr. Shirley
Mr. Simmonds
Mr. Edward Smith
Mr. Jofeph Steel
Mr. R. H. H. Steel
Mr. Strong
Mr. Turnbull
Mr. Tufon
Mr. Ware
Mr. William Warner
Mr. Witham
Mr. Wood
Mr. Woodward
Mr. Wye
Mr. G. W. Young
Mr. Jonathan Young

